# Comparing verbal autopsy cause of death findings as determined by physician coding and probabilistic modelling: a public health analysis of 54 000 deaths in Africa and Asia

**DOI:** 10.7189/jogh.05.010402

**Published:** 2015-06

**Authors:** Peter Byass, Kobus Herbst, Edward Fottrell, Mohamed M. Ali, Frank Odhiambo, Nyaguara Amek, Mary J. Hamel, Kayla F. Laserson, Kathleen Kahn, Chodziwadziwa Kabudula, Paul Mee, Jon Bird, Robert Jakob, Osman Sankoh, Stephen M. Tollman

**Affiliations:** 1 WHO Collaborating Centre for Verbal Autopsy, Umeå Centre for Global Health Research, Umeå University, Sweden; 2 Medical Research Council/Wits University Rural Public Health and Health Transitions Research Unit (Agincourt), School of Public Health, Faculty of Health Sciences, University of the Witwatersrand, Johannesburg, South Africa; 3 IMMPACT, Institute of Applied Health Sciences, School of Medicine and Dentistry, University of Aberdeen, Aberdeen, UK; 4 Africa Centre for Health and Population Studies, University of KwaZulu–Natal, KwaZulu–Natal, South Africa; 5 INDEPTH Network, Accra, Ghana; 6 UCL Institute for Global Health, University College London, London, UK; 7 Eastern Mediterranean Regional Office, World Health Organization, Cairo, Egypt; 8 KEMRI/CDC Research and Public Health Collaboration, Kisumu, Kenya; 9 CDC Malaria Branch, Atlanta, GA, USA; 10 CDC Center for Global Health, Atlanta, GA, USA; 11 School of Informatics, City University London, London, UK; 12 World Health Organization, Geneva, Switzerland; 13 Hanoi Medical University, Hanoi, Vietnam

## Abstract

**Background:**

Coverage of civil registration and vital statistics varies globally, with most deaths in Africa and Asia remaining either unregistered or registered without cause of death. One important constraint has been a lack of fit–for–purpose tools for registering deaths and assigning causes in situations where no doctor is involved. Verbal autopsy (interviewing care–givers and witnesses to deaths and interpreting their information into causes of death) is the only available solution. Automated interpretation of verbal autopsy data into cause of death information is essential for rapid, consistent and affordable processing.

**Methods:**

Verbal autopsy archives covering 54 182 deaths from five African and Asian countries were sourced on the basis of their geographical, epidemiological and methodological diversity, with existing physician–coded causes of death attributed. These data were unified into the WHO 2012 verbal autopsy standard format, and processed using the InterVA–4 model. Cause–specific mortality fractions from InterVA–4 and physician codes were calculated for each of 60 WHO 2012 cause categories, by age group, sex and source. Results from the two approaches were assessed for concordance and ratios of fractions by cause category. As an alternative metric, the Wilcoxon matched–pairs signed ranks test with two one–sided tests for stochastic equivalence was used.

**Findings:**

The overall concordance correlation coefficient between InterVA–4 and physician codes was 0.83 (95% CI 0.75 to 0.91) and this increased to 0.97 (95% CI 0.96 to 0.99) when HIV/AIDS and pulmonary TB deaths were combined into a single category. Over half (53%) of the cause category ratios between InterVA–4 and physician codes by source were not significantly different from unity at the 99% level, increasing to 62% by age group. Wilcoxon tests for stochastic equivalence also demonstrated equivalence.

**Conclusions:**

These findings show strong concordance between InterVA–4 and physician–coded findings over this large and diverse data set. Although these analyses cannot prove that either approach constitutes absolute truth, there was high public health equivalence between the findings. Given the urgent need for adequate cause of death data from settings where deaths currently pass unregistered, and since the WHO 2012 verbal autopsy standard and InterVA–4 tools represent relatively simple, cheap and available methods for determining cause of death on a large scale, they should be used as current tools of choice to fill gaps in cause of death data.

“Civil registration and vital statistics don’t quicken everyone’s pulse.” So wrote Richard Horton [1] in summarising the first Global Summit on Civil Registration and Vital Statistics (CRVS), held in Bangkok in April 2013. But, as was clear from that meeting, global understanding of public health depends on having an adequately comprehensive overview of cause–specific mortality patterns at the population level. Counting people and their life events is a big part of what needs to be done more effectively and comprehensively [2]; added to that is the need to attribute cause to deaths in a systematic, rapid, consistent and cost–effective way.

Unsatisfactory progress in CRVS over recent decades lay at the heart of the four major objectives of the WHO Commission on Information and Accountability for Women’s and Children’s Health (COIA) [3]. Accountability at every level ultimately depends on effectively counting individuals, and then making good use of those data. Implementation of COIA’s recommendations was entrusted to an independent Evidence Review Group (iERG), which, in its 2013 report [4], acknowledged that COIA’s recommendation on enhancing CRVS will be “difficult or impossible to achieve” by the target date of 2015. Instead, iERG now recommends making effective CRVS a post–2015 development target. While there are evidently many practical obstacles to achieving reliable CRVS on a global scale, one prerequisite component is the availability of fit–for–purpose tools for registering deaths and assigning cause of death. Such tools must be openly accessible, and be capable of delivering consistent and systematic mortality data in a timely and cost–effective manner.

Verbal autopsy (VA; interviewing a care–giver, relative or witness after a death, and using the interview material to determine cause of death) is seen as an essential interim approach for filling in some of the gaps in global knowledge on cause–specific mortality [5], which can otherwise only be estimated [6]. Although, in the long–term, one might hope for universal physician certification of deaths, undertaken methodically and rigorously, this will not be the case for most deaths in Africa and Asia for the foreseeable future. The immediate public health concern therefore is to establish VA methods for determining cause of death which are readily applicable on a large scale (including in routine CRVS processes) and provide sufficient detail for effective health planning.

Verbal autopsy interview material has been collected in a variety of ways, and then interpreted into cause of death data by various methods. There has therefore been substantial methodological heterogeneity involved, which can magnify existing uncertainties over cause–specific mortality. The World Health Organization (WHO) released a new standard for VA data collection together with a revised set of cause of death categories (with equivalence to the International Classification of Diseases version 10 [ICD–10]) in 2012 [7]. The process undertaken to streamline previous VA approaches into the new 2012 WHO VA standard is described in detail elsewhere [5].

Ways of interpreting VA data essentially fall into physician consideration of individual cases (physician–coded verbal autopsy, PCVA) or various mathematical approaches to automated processing of VA data. PCVA has been a *de facto* standard in many research settings, although associated details of methods and validity have not always been well established [8] other than in specific studies of hospital–based deaths. PCVA is generally considered too slow and expensive for routine CRVS implementation, apart from the disadvantage of consuming often scarce physician time. A number of approaches to automated processing have been tried over the last decade or so; the currently most widely used is the InterVA suite of models that apply Bayesian probabilistic modelling, and which have been in the public domain in various versions since 2005 (at www.interva.net) [9]. Corresponding to the release of the 2012 WHO VA standard, InterVA–4 was released in 2012, incorporating exactly the same range of input and output parameters as specified by WHO [10].

Nevertheless, monitoring cause–specific mortality is a long–term process, and so much of the existing VA material which is archived in various places reflects earlier standards and variations. It will be some time yet before any substantial body of VA data originally collected according to the provisions of the 2012 WHO VA standard becomes available. Our aim in this paper is to take VA archives from a variety of pre–2012 sources, which have also been assessed by PCVA, convert them insofar as is possible into the 2012 WHO format, and compare the PCVA and InterVA–4 findings. Our objective is primarily methodological. Rather than attempting to illuminate specific epidemiological findings, we evaluate the consistency between applying the 2012 WHO VA standard and the corresponding InterVA–4 model to existing secondary data, and compare this with the primary physician–coded findings from the same data. The underlying consideration is the public health consistency and relevance of the two approaches – InterVA–4 and PCVA – as a source of information for health planning in regions where routine cause–specific mortality data are scarce. Many national and regional public health practitioners are posing the question as to whether they can reasonably rely on verbal autopsy surveillance with automated methods for assigning cause of death to monitor mortality patterns in the populations they serve: this study aims to answer that question.

## DATA SOURCES AND METHODS

For the purposes of this comparison, we have selected several VA data sets for secondary analyses on grounds of availability, variety of original VA procedures, coverage of diverse geographic locations and population groups, and with well–established local PCVA procedures. PCVA procedures varied slightly between sites, but for every site the consensus “main” or “underlying” cause was used here. The sources and characteristics of the data are shown in **Table 1**. Data were sourced from Afghanistan, Bangladesh, Ghana, Kenya and South Africa. The original sources were of two main types, Demographic and Household Surveys (DHS) [17] and INDEPTH Network Health and Demographic Surveillance Systems (HDSS) [18] but there were also local variations in the details of VA procedures used within these two groupings. The locations also cover a wide range of HIV and malaria prevalences, which are the two causes of death which vary most markedly geographically. The two sites in South Africa are only 600 km apart and share a number of characteristics, but used different VA procedures. All of the PCVA results were reported using ICD–10 codes, enabling direct comparison with the InterVA–4 outputs using the WHO 2012 ICD–10 cause category definitions.

**Table 1:** Characteristics of the six data sources used

Source	Type of data	Location	Population group	Period deaths occurred	Verbal autopsy instrument	Deaths covered	Reference
Afghanistan	DHS	National cluster sample survey	Entire	2005–2010	DDHS form	3349	[11]
Bangladesh	DHS	National cluster sample survey	Women aged 12 to 49 y	1997–2001	DHS form	928	[12]
Ghana	DHS	National cluster sample survey	Women aged 12 to 49 y	2002–2007	DHS form	4203	[13]
Kenya	INDEPTH HDSS	Surveillance site in Siaya County	Entire	2003–2010	Adapted INDEPTH form	21 236	[14]
South Africa A	INDEPTH HDSS	Surveillance site in Bushbuckridge	Entire	1992–2010	Locally adapted form	10 139	[15]
South Africa B	INDEPTH HDSS	Surveillance site in Kwa–Zulu Natal	Entire	2000–2011	Adapted INDEPTH form	14 327	[16]

DHS – Demographic and Health Survey, HDSS – Health and Demographic Surveillance System

Stata command files were created for each site to extract as many as possible of the 2012 WHO InterVA indicators for each case (possible indicators total 244 across all age–sex groups, with the number of applicable questions for any particular death ranging from 54 to 181) from the various VA data sets. VA records which did not contain any symptom data (ie, only identification and background indicators) or which did not include valid age and sex details were excluded. The VA data from each source were then processed using InterVA–4 (version 4.02) and the cause of death outputs processed into cause–specific mortality fractions (CSMF) as previously described [10]. PCVA outputs, specified as ICD–10 codes, were categorised into the 2012 WHO VA cause of death groups for comparative purposes, using the conversion table specified in the WHO documentation. Age–groups corresponding to WHO 2012 categories (0–28 days, 1–11 months, 1–4 years, 5–14 years, 15–49 years, 50–64 years and 65+ years) were used as the basis for analysis. Because of inherent uncertainty at the individual level in differentiating in many cases between the 01.03 HIV/AIDS and 01.09 pulmonary TB cause categories, both for InterVA–4 and PCVA, comparisons are presented with those categories separate and combined.

CSMFs were calculated for each source and cause of death, separately for InterVA–4 and PCVA findings. Concordance between InterVA–4 and PCVA CSMFs was measured using Lin’s concordance correlation coefficient [19], corrected and implemented for Stata [20]. As an alternative metric for assessing the equivalence of CSMFs from InterVA–4 and PCVA findings, we used the Wilcoxon matched–pairs signed ranks test and its two one–sided tests (TOST) variant for stochastic equivalence, with epsilon set to 3, as implemented for Stata [21]. Ratios of CSMFs according to InterVA–4 and PCVA, by source, age–sex group and cause, were calculated together with 99% CIs, according to the Katz adjusted log method which permits the estimation of intervals around ratios where one side is zero [22]. CIs were calculated at the 99% level as hundreds of separate ratios were assessed. The objective of calculating these CIs was not so much for the sake of demonstrating statistical significance, but rather to identify particular causes and age–sex groups for which the CSMF ratios between interpretations by InterVA–4 and physicians were appreciably lower or higher than might be expected by chance, taking into account the number of cases involved.

No specific ethical clearance was required for this study, which relied solely on the analysis of existing secondary data, without individually identifiable information. For the Kenya data set, in Kisumu, following cultural customs, compound heads provide written consent for all compound members to participate in the HDSS activities. Any individual can refuse to participate at any time. The Kisumu HDSS protocol and consent procedures, including surveillance and VA, were approved by KEMRI and CDC Institutional Review Boards annually. For the South Africa A data set, surveillance–based studies in the Agincourt subdistrict were reviewed and approved by the Committee for Research on Human Subjects (Medical) of the University of the Witwatersrand, Johannesburg, South Africa (protocol M960720, renewed). Informed consent was obtained at the individual and household levels at every follow–up visit, whereas community consent from civic and traditional leadership was secured at the start of surveillance and reaffirmed from time to time. For the South Africa B data set, ethical approval for the Africa Centre Demographic Surveillance was provided by the University of Kwa–Zulu–Natal Bio–Medical Research Ethics Committee (protocol E009/00).

## RESULTS

**Figure 1:**
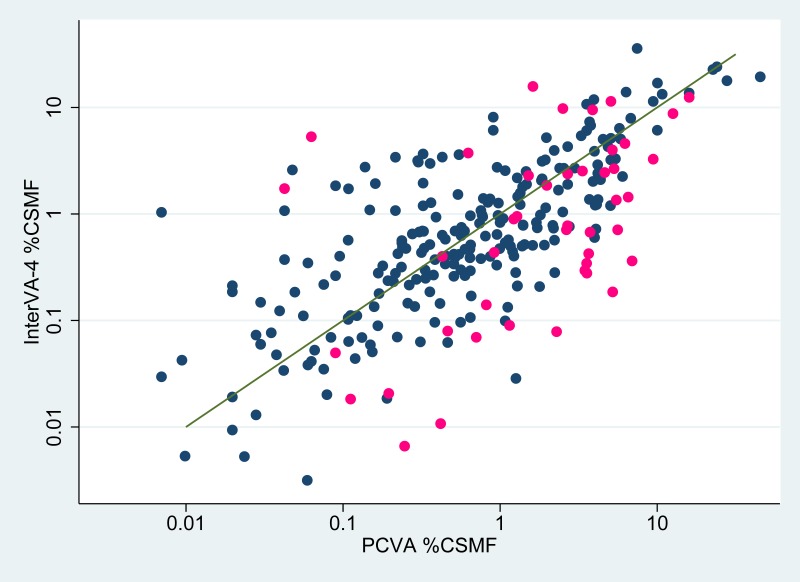
Correlation for cause–specific mortality fractions (CSMF) for WHO 2012 causes of death from six data sources, as determined by InterVA–4 [10] and physician–coded verbal autopsy (PCVA) for 54 182 verbal autopsies, against the line of equivalence. Pink markers represent residual cause categories; blue markers represent specific causes.

Over the total of 54 182 VA records analysed, **Table 2** shows concordance correlation coefficients by data source and by age–group, both for the basic outputs and with the HIV and TB categories combined for sub–Saharan Africa. **Figure 1** shows, for each WHO 2012 cause category and over all the six sources, a scatter plot of CSMFs from both InterVA–4 and PCVA interpretations. The corresponding concordance correlation coefficient was 0.831 (95% CI 0.751–0.911), and this increased to 0.974 (95% CI 0.961–0.987) when the 01.03 HIV/AIDS and 01.09 pulmonary TB cause categories were combined for sub–Saharan Africa. **Table 3** shows results from the alternative Wilcoxon’s metric for equivalence between CSMFs. Equivalence is represented by the large p values for the standard Wilcoxon’s signed rank test (not permitting rejection of the null hypothesis of no difference) together with significant p values indicating that differences lay within the equivalence range.

**Table 2:** Concordance correlation coefficients (CCC) for InterVA–4 [10] and physician–coded verbal autopsy (PCVA) interpretations of 54 182 verbal autopsies from 6 sources

	Deaths	Basic data		HIV/AIDS and pulmonary TB categories combined	
		**CCC**	**95% CI**	**CCC**	**95% CI**
**Overall**	54 182	0.831	0.751–0.911	0.974	0.961–0.987
Source:					
Afghanistan	3349	0.625	0.464–0.787	–	–
Bangladesh	928	0.720	0.580–0.860	–	–
Ghana	4203	0.665	0.509–0.821	0.751	0.631–0.871
Kenya	21 236	0.854	0.785–0.923	0.923	0.885–0.960
South Africa A	10 139	0.912	0.868–0.956	0.947	0.922–0.972
South Africa B	14 327	0.588	0.415–0.760	0.990	0.985–0.995
**Age–group:**					
0–28 d	1678	0.529	0.258–0.801	0.529	0.258–0.801
1–11 mo	5070	0.813	0.722–0.904	0.810	0.713–0.908
1–4 y	5123	0.886	0.824–0.948	0.909	0.857–0.961
5–14 y	1734	0.828	0.733–0.922	0.888	0.826–0.949
15–49 y	24 478	0.771	0.663–0.880	0.991	0.986–0.996
50–64 y	6239	0.784	0.667–0.902	0.981	0.969–0.993
65+ years	9860	0.846	0.760–0.931	0.895	0.835–0.956

CI – confidence interval, TB - tuberculosis

**Table 3:** Statistical analysis of ranked cause-specific mortality fractions, overall and by source, using the Wilcoxon matched–pairs signed ranks test and its two one–sided tests variant for stochastic equivalence

Source	Wilcoxon matched pairs signed ranks (*P*)	Two one–sided tests variant for stochastic equivalence (ϵ = 3) p_low_, p_high_
Overall	0.187	0.001, 0.047
Afghanistan	0.808	0.001, 0.003
Bangladesh	0.870	0.002, 0.001
Ghana	0.358	0.001, 0.007
Kenya	0.607	0.001, 0.007
South Africa A	0.262	0.001, 0.030
South Africa B	0.509	0.001, 0.010

Graphical presentations for each source separately, in a similar format to **Figure 1**, are available in **Online Supplementary Document(Online Supplementary Document)**, which also show WHO 2012 cause categories. **Table 4** shows the CSMF for each WHO 2012 cause category and site, as determined by InterVA–4 and PCVA.

**Table 4:** Cause–specific mortality fractions from 54 182 verbal autopsies, by WHO 2012 virtual autopsy cause category and data source

Cause of death	Data source											
	**Afghanistan** **(3349 deaths)**		**Bangladesh** **(928 deaths)**		**Ghana** **(4203 deaths)**		**Kenya** **(21 236 deaths)**		**South Africa A** **(10 139 deaths)**		**South Africa B** **(14 327 deaths)**	
	InterVA–4*	**PCVA**	**InterVA–4**	**PCVA**	**InterVA–4**	**PCVA**	**InterVA–4**	**PCVA**	**InterVA–4**	**PCVA**	**InterVA–4**	**PCVA**
01.01 Sepsis (non–obstetric)	0.26	0.09			0.01		0.24	0.19	0.19	0.02	0.02	
01.02 Acute resp. infect, incl. pneumonia	11.41	9.44	3.41	0.22	0.74	2.19	13.95	6.34	11.86	3.96	6.37	5.75
01.03 HIV/AIDS related death	0.89		0.12		22.73	22.65	17.85	27.82	24.09	24.01	19.41	45.33
01.04 Diarrhoeal diseases	5.06	5.85	1.37	4.20	1.20	5.04	2.41	4.19	2.02	3.91	0.57	2.22
01.05 Malaria	0.40	1.22	0.96	0.65	2.25	6.02	13.66	15.98	0.50	1.38	0.42	0.22
01.06 Measles	0.72						0.69	0.32	0.07		0.05	
01.07 Meningitis and encephalitis	2.51	1.46	1.52	0.54		3.55	0.76	2.76	0.51	1.91	1.04	2.51
01.08, 10.05 Tetanus							0.01	0.02			0.01	
01.09 Pulmonary tuberculosis	10.73	3.55	6.79	3.77	7.34	3.71	13.33	10.76	16.98	10.04	35.85	7.45
01.10 Pertussis	0.13						0.03		0.26		0.03	
01.11 Haemorrhagic fever	0.06						0.01		0.01	0.01		
01.99 Other and unspecified infect dis	1.35	5.49	0.34	3.56	0.19	5.21	0.95	1.28	0.68	3.74	0.14	0.82
02.01 Oral neoplasms	0.35	0.06	1.19	0.32	0.46	0.00	0.14		0.21	0.02	0.11	0.06
02.02 Digestive neoplasms	2.90	4.18	6.07	3.56	3.42	0.43	1.90	1.47	2.75	0.96	1.40	0.78
02.03 Respiratory neoplasms	1.84	0.09	1.95	0.32	2.59	0.05	1.72	0.11	0.56	0.24	1.93	0.16
02.04 Breast neoplasms	0.47	0.60	2.55	1.08	2.18	1.28	0.07	0.22	0.68	0.31	0.23	0.21
02.05, 02.06 Reproductive neoplasms M,F	0.49	0.24	4.29	2.69	3.61	0.55	0.33	0.95	0.98	1.80	0.98	0.77
02.99 Other and unspecified neoplasms	2.53	3.34	2.45	4.63	0.28	3.57	2.29	1.52	1.85	1.98	0.90	1.22
03.01 Severe anaemia	0.78		1.08	0.22	0.05		0.28	2.23	0.09		0.24	
03.02 Severe malnutrition	3.95	2.21	0.68		0.04		0.72	4.07	0.50	1.16	0.39	0.52
03.03 Diabetes mellitus	1.21	4.03	1.39	0.86	0.13	1.12	0.57	1.13	1.80	1.39	1.68	2.35
04.01 Acute cardiac disease	0.83	1.70	1.90	2.69	0.47	0.64	0.37	0.04	0.43	0.32	0.44	1.20
04.03 Sickle cell with crisis					0.18		0.27	0.38				
04.02 Stroke	4.28	4.87	7.92	6.79	1.23	4.12	1.23	1.34	2.10	4.36	3.30	5.42
04.99 Other and unspecified cardiac dis.	3.27	9.44	9.49	3.88	4.58	6.23	3.74	0.63	2.66	5.32	3.99	5.19
05.01 Chronic obstructive pulmonary dis.	1.58	1.34	0.10	0.11	0.24	0.00	0.60	3.99	2.76	0.14	1.28	0.36
05.02 Asthma	1.29	0.84	0.78	1.40	6.11	0.90	0.34	0.45	0.69	0.33	0.69	0.52
06.01 Acute abdomen	2.98	0.36	3.66	0.32	8.12	0.90	3.07	0.30	1.09	0.15	1.04	0.01
06.02 Liver cirrhosis	0.75	0.57	3.88	3.99	0.78	2.17	0.63	0.57	0.52	1.43	0.28	1.26
07.01 Renal failure	0.26	0.51	3.23	1.94	1.27	0.98	0.47	0.99	0.14	0.41	0.51	0.65
08.01 Epilepsy	0.40	0.87	1.46	1.29	0.03	1.26	0.17	0.65	0.30	0.56	0.40	0.45
98 Other and unspecified NCD	0.78	2.69	2.38	2.69	0.36	6.92	1.73	0.04	0.71	2.64	0.08	2.29
10.06 Congenital malformation	0.51	1.61		0.11			0.07	0.13	0.06	0.46	0.15	0.26
10.01 Prematurity	2.14	1.85					0.10	0.56	0.82	0.74	0.10	0.38
10.02 Birth asphyxia	3.17	0.30					0.93	0.39	0.53	0.24	0.24	0.29
10.03 Neonatal pneumonia	5.21	1.97					1.07	0.04	0.65	0.28	0.47	0.25
10.04 Neonatal sepsis	1.37	3.70					0.21	1.29	0.12	0.04	0.07	0.03
10.99 Other and unspecified neonatal CoD	1.44	6.54					0.40	0.43	0.08	0.46	0.02	0.11
12.01 Road traffic accident	2.70	2.99	0.28	0.22	2.06	1.83	0.42	0.51	2.43	2.69	2.69	2.39
12.02 Other transport accident		0.06				0.02				0.01		0.70
12.03 Accid. fall	0.64	0.96		0.11	0.42	0.55	0.22	0.08		0.10	0.04	0.06
12.04 Accid. drowning and submersion	0.62	0.81	0.11	0.65	0.30	0.33	0.33	0.18	0.14	0.29	0.25	0.34
12.05 Accid. expos to smoke, fire & flame	0.26	0.60	0.29	0.65	0.09	0.17	0.22	0.26	0.37	0.38	0.28	0.17
12.06 Contact with venomous plant/animal	0.34	0.51	0.97	0.97	0.40	0.52	0.11	0.12		0.09	0.08	0.03
12.10 Exposure to force of nature		0.06		0.32		0.12	0.04	0.01		0.15		0.03
12.07 Accid. poisoning and noxious subs	0.04	0.12	0.03		0.02	0.19	0.06	0.31	0.13	0.16	0.05	0.15
12.08 Intentional self–harm	0.48	0.33	6.12	10.02	0.40	0.10	0.32	0.24	0.79	1.40	0.94	0.77
12.09 Assault	3.13	1.85	0.38	0.75	0.52	0.36	0.69	0.59	2.69	2.54	5.14	5.07
12.99 Other and unspecified external CoD	0.29	3.46		1.83		0.31	0.09	1.15	0.44	0.92	0.07	0.70
09.01 Ectopic pregnancy			0.11	0.11	0.63	0.43	0.01		0.01	0.02	0.03	0.01
09.02 Abortion–related death	0.06	0.03	0.54	1.08	1.14	1.95	0.03	0.08		0.06	0.01	0.03
09.03 Pregnancy–induced hypertension	0.58	0.45	5.04	4.53	0.21	1.28	0.05	0.04	0.06	0.11	0.11	0.11
09.04 Obstetric haemorrhage	0.91	1.05	3.23	5.06	5.43	3.28	0.18	0.17	0.18	0.05	0.07	0.08
09.05 Obstructed labour	0.06	0.15	0.10	1.08	0.39	0.64						
09.06 Pregnancy–related sepsis	0.15	0.03	1.08	0.75	0.83	1.00	0.05	0.07	0.02	0.08	0.03	0.04
09.07 Anaemia of pregnancy	0.04	0.06	0.74	1.72	0.21	1.78	0.04		0.02	0.02	0.03	
09.08 Ruptured uterus			0.57	0.11	0.19	0.36		0.01				0.01
09.99 Other and unspecified maternal CoD	0.01	0.42	0.71	5.60	0.42	3.66	0.05	0.09	0.01	0.25	0.02	0.20
99 Indeterminate	11.41	5.08	8.76	12.61	15.77	1.62	9.77	2.51	12.45	15.99	5.32	0.06
Overall	100.00	100.00	100.00	100.00	100.00	100.00	100.00	100.00	100.00	100.00	100.00	100.00

VA – verbal autopsy, PCVA – physician–coded verbal autopsy, M – male, F – female, CoD – cause of death

*InterVA–4 software [10].

Using the CSMFs shown in **Table 3** for each cause and source, CSMF ratios InterVA–4:PCVA were calculated with 99% confidence intervals as a basis for comparison. These are tabulated fully in Additional File 1. Of the 320 source/cause comparisons that were made, 171 (53.4%) of these ratios were not significantly different from unity at the 99% level.

CSMFs were similarly calculated by age–group and sex, across all sources. These results, in a similar format to **Table 2**, are shown in **Online Supplementary Document(Online Supplementary Document)**. A further table in **Online Supplementary Document(Online Supplementary Document)** shows CSMF ratios InterVA–4:PCVA, with 99% confidence intervals, for each cause and age–sex group, over all data sources. Of the 530 age–sex/cause comparisons that were made, 329 (62.1%) of these ratios were not significantly different from unity at the 99% level.

## DISCUSSION

Our results show a generally good level of agreement between the InterVA–4 and PCVA approaches to the interpretation of this large VA data set, over diverse populations. There are some important differences, discussed below, but nevertheless the two approaches achieved good public health equivalence, meaning that taking public health and health planning measures on the basis of either source would lead to similar conclusions. This concept of “public health equivalence” is very important in interpreting these findings. Development of VA methods in recent years has led to a situation in which public health practitioners in countries where deaths are not routinely registered with causes are posing important practical questions. They need to know whether they can reasonably rely on modern VA methods with automated interpretation to provide policy–relevant information on mortality patterns in a cost–effective manner. This is not just a matter of identifying major causes of death – it is equally critical, for example, to monitor causes that have become rare, such as measles, in order to be sure of the continued effectiveness of vaccination programmes. Previous work [23,24] has shown that InterVA–4 can be effectively operationalised at much lower cost than PCVA; here we demonstrate its functional equivalence to PCVA.

It is critical to realise that neither InterVA–4 nor PCVA, nor indeed the underlying VA data to which they have been applied, necessarily represent absolute truth (whatever that may be) in terms of cause of death. Cause of death assignment is, at best, a mixture of science and judgement [25]. There is an extensive literature on comparisons between different methods for determining cause of death, which show substantial inter–method variations. A review of clinical cause of death assignment and post–mortem findings found rates of discrepancies ranging from 30% to 63% across the 18 included studies [26]. Pre–mortem CT imaging has been evaluated as only able to correctly identify 66% of post–mortem examination causes of death [27]. In South Africa, an autopsy series on miners found that 51% of respiratory infections diagnosed at autopsy had not been noted clinically [28]. There is a clear need to improve future VA methods by validating causes of death directly against post–mortem findings, but that is a major undertaking given the widespread lack of autopsies undertaken in Africa and Asia [29]. Against this background of high discrepancy rates between post–mortem findings and other methods of assigning cause of death, the relatively good agreement between PCVA and InterVA–4 findings here is encouraging, even though both might differ from post–mortem findings if those were available.

Attempts have been made to validate VA approaches in specific studies with hospital or laboratory data [30]. Some specific causes of death are amenable to this approach, for example by using particular data sets where *ante–mortem* HIV or sickle–cell status is documented [31,32]. A study from the Population Health Metrics Research Consortium recruited tertiary facility deaths across a range of hospital–assigned pre–determined causes, which were followed up with VA interviews [33]. This data set was used to build new models for assigning cause of death, which were then tested together with other models and physician assigned causes in the same data set. Unsurprisingly, models built within this data set performed better in relation to the hospital causes than either other models or physicians [34]. Further bench–testing of VA interpretation models showed roughly equivalent performance across various models when compared to PCVA as the reference standard [35]. By defining performance in relation to PCVA, however, these evaluations precluded comparison of public health consistency between models and physicians.

Analytical methods for comparing cause of death assignment are not entirely straightforward, because of the general uncertainty associated with cause of death, the interplay between precipitating and underlying causes, and the nature of the data. Here we have concentrated on comparing CSMFs, since that is the primary outcome of interest from cause of death data in public health. The concordance correlation coefficients and rank equivalence tests used here present accessible and convenient summary measures of how CSMFs from two different sources compared. For individual cause comparisons by factors such as source, age–group and sex, the ratio between CSMFs by the two methods provides insight on specific aspects for comparison, and the confidence interval of that ratio is informative in deciding whether or not differences are due to chance. It has been suggested that comparisons between cause of death methods should be corrected for chance agreement, which is more likely to occur in common causes [36]. However, from a public health perspective this is not necessarily appropriate, since in practice agreement is generally accepted irrespective of the possibility that it was derived by chance.

The overall size and geographic diversity of the data presented here are important attributes. These VA data were not collected under carefully controlled and standardised procedures in order to minimise real–life sources of variation; this is a major strength of this study. The sources deliberately included a mix of high and low HIV and malaria settings, which are the two causes of highest variation in CSMF findings between specific settings. In any cause of death data, a relatively small number of more common causes account for the majority of the deaths, followed by many causes accounting for small fractions in the remainder. Consequently it is only possible to evaluate cause of death methods thoroughly in data sets which are large enough to include realistic numbers of rarer causes. Globally, most unrecorded deaths occur in Africa and Asia, which are therefore the regions where VA methods are most urgently needed, and which are represented in these data. It must also be noted that inevitably none of these archived data sets were originally collected under the WHO 2012 VA standard, and hence some degree of inter–site variation may have been introduced in the process of extracting the necessary VA indicator data.

One commonly contentious area in terms of cause of death is the interaction between HIV/AIDS and pulmonary TB. Three of the six data sources included substantial numbers of HIV/AIDS deaths during the periods covered by these data, and both InterVA–4 and PCVA findings reflected that. A validation study for InterVA–4 in relation to HIV sero–status showed high specificity for HIV/AIDS as a cause of death (ie, relatively few false–positive HIV/AIDS cause assignments) but also showed considerably elevated mortality rates among sero–positives for causes such as pneumonia and pulmonary tuberculosis [31]. Although ICD–10 coding in principle requires the use of codes B20–B24 where HIV and co–infections are involved, the extent to which this can reliably be implemented using VA methods is debatable, particularly if VA respondents are unaware of the HIV status of the deceased. In these analyses, there are clear differences between the two South African sources in this respect, with appreciably different proportions of deaths assigned as HIV/AIDS or tuberculosis. Conversely, in low HIV/AIDS or malaria settings, physicians may be reluctant to assign deaths to those causes. For example in the Afghan data set, where very few HIV/AIDS deaths might be expected, HIV/AIDS was explicitly mentioned in four VA interviews, but this was not reflected in the PCVA results, which never assigned HIV/AIDS as a cause of death.

Any cause of death assignment process, at the individual level, will involve some degree of uncertainty. Formal procedures for assigning cause of death, for example in official death certificates, do not generally capture this uncertainty, but require the certifier to make a clear choice between possible causes [8]. Even if two certifiers are required to assess a case independently, as is often practised in PCVA, agreement does not necessarily constitute truth. One factor that emerges clearly from these analyses is that in the PCVA findings there is a greater tendency for physicians to choose chapter residual categories (pink markers in **Figure 1**), rather than specific causes (blue markers in **Figure 1**). This is evident from most of the pink markers lying below the line of equivalence, and is probably an expression of PCVA uncertainty. This was particularly evident in the neonatal age group, in addition to cross–over between neonatal sepsis and pneumonia categories, as seen in **Online Supplementary Document(Online Supplementary Document)**, Table s2, resulting in the lower correlation observed for neonates. On the other hand, InterVA–4, by using a probabilistic model, computes a residual uncertainty for each case which is then expressed as an indeterminate component. By expressing uncertainty in this way, CSMFs for indeterminate causes may be greater according to InterVA–4.

## CONCLUSIONS

Given the inherent difficulties and uncertainties involved in assigning cause of death, and the urgent need to implement large–scale, cost–effective CRVS procedures that include cause of death, it is clear that the priority for the foreseeable future in many low– and middle–income countries will be to undertake VA with automated cause of death assignment. We have shown here, using a large and diverse data set, that there is a strong correlation between in–country PCVA findings and outputs from the freely available InterVA–4 model, over a wide range of settings. Whilst accepting that neither PCVA nor InterVA–4 results necessarily represent absolute truth, and that there is a continuing search for improved methods for assigning causes of death, the use of InterVA–4 represents a low–resource and highly consistent strategy, which is a major advance on knowing almost nothing about cause of death profiles in many populations. The diversity of cause of death profiles which InterVA–4 produces across the various sources clearly demonstrates that a standard model can be used successfully over a wide range of settings. InterVA–4, and the WHO 2012 VA standard with which it is fully compatible, should therefore be used as the currently available tools of choice for filling gaps in cause–specific CRVS data.

## References

[1] Horton R (2013). Offline: I find such people intolerable.. Lancet.

[2] Byass P (2012). The UN needs joined–up thinking on vital registration.. Lancet.

[3] World Health Organization. Commission on Information and Accountability for Women’s and Children’s Health: Keeping Promises, Measuring Results. Geneva: WHO, 2011. Available: http://www.everywomaneverychild.org/images/content/files/accountability_commission/final_report/Final_EN_Web.pdf. Accessed: 5 January 2015.

[4] World Health Organization. Every Woman, Every Child: Strengthening Equity and Dignity through Health: the second report of the independent Expert Review Group (iERG) on Information and Accountability for Women’s and Children’s health. Geneva: WHO, 2013. Available: http://apps.who.int/iris/bitstream/10665/85757/1/9789241505949_eng.pdf. Accessed: 5 January 2015.

[5] Leitao J, Chandramohan D, Byass P, Jakob R, Bundhamcharoen K, Choprapawon C (2013). Revising the WHO verbal autopsy instrument to facilitate routine cause–of–death monitoring.. Glob Health Action..

[6] Byass P, de Courten M, Graham WJ, Laflamme L, McCaw–Binns A, Sankoh OA (2013). Reflections on the global burden of disease 2010 estimates.. PLoS Med.

[7] World Health Organization. Verbal autopsy standards: the 2012 WHO verbal autopsy instrument. Geneva: WHO, 2012. Available: http://www.who.int/healthinfo/statistics/WHO_VA_2012_RC1_Instrument.pdf. Accessed: 5 January 2015.

[8] Byass P (2011). The democratic fallacy in matters of clinical opinion: implications for analysing cause–of–death data.. Emerg Themes Epidemiol.

[9] Byass P, Fottrell E, Huong DL, Berhane Y, Corrah T, Kahn K (2006). Refining a probabilistic model for interpreting verbal autopsy data.. Scand J Public Health.

[10] Byass P, Chandramohan D, Clark SJ, D'Ambruoso L, Fottrell E, Graham WJ (2012). Strengthening standardised interpretation of verbal autopsy data: the new InterVA–4 tool.. Glob Health Action.

[11] Afghan Public Health Institute. Afghanistan Mortality Survey 2010. Calverton, Maryland, USA, 2011. Available: http://measuredhs.com/pubs/pdf/FR248/FR248.pdf. Accessed: 5 January 2015.

[12] National Institute of Population Research and Training. Bangladesh maternal health services and maternal mortality survey 2001. Calverton, Maryland, USA, 2003. Available: http://measuredhs.com/pubs/pdf/FR142/FR142.pdf. Accessed. 5 January 2015.

[13] Ghana Statistical Service. Ghana maternal health survey 2007. Accra, Ghana: GSS, 2009. Available: http://measuredhs.com/pubs/pdf/FR227/FR227.pdf. Accessed: 5 January 2015.

[14] van Eijk AM, Adazu K, Ofware P, Vulule J, Hamel M, Slutsker L (2008). Causes of deaths using verbal autopsy among adolescents and adults in rural western Kenya.. Trop Med Int Health.

[15] Kahn K, Collinson MA, Gómez–Olivé FX, Mokoena O, Twine R, Mee P (2012). Profile: Agincourt health and socio–demographic surveillance system.. Int J Epidemiol.

[16] Herbst AJ, Mafojane T, Newell M-L (2011). Verbal autopsy–based cause–specific mortality trends in rural KwaZulu–Natal, South Africa, 2000–2009.. Popul Health Metr.

[17] Corsi DJ, Neuman M, Finlay JE, Subramanian SV (2012). Demographic and health surveys: a profile.. Int J Epidemiol.

[18] Sankoh O, Byass P (2012). The INDEPTH Network: filling vital gaps in global epidemiology.. Int J Epidemiol.

[19] Lin LI-K (1989). A concordance correlation coefficient to evaluate reproducibility.. Biometrics.

[20] Cox NJ, Steichen T. CONCORD: Stata module for concordance correlation. Statistical Software Components S404501, Boston College Department of Economics, Boston; 2000. Available: http://ideas.repec.org/c/boc/bocode/s404501.html. Accessed: 8 July 2014.

[21] Dinno A. tost: Two one–sided tests of equivalence. Stata software package. Available: http://www.doyenne.com/stata/tost.html. Accessed: 6 September 2014.

[22] Fagerlund MW, Lydersen S, Laake P (2011). Recommended confidence intervals for two independent binomial proportions.. Stat Methods Med Res.

[23] Byass P, Kahn K, Fottrell E, Collinson MA, Tollman SM (2010). Moving from data on deaths to public health policy in Agincourt, South Africa: Approaches to analysing and understanding verbal autopsy findings.. PLoS Med.

[24] Godefay H, Abrha A, Kinsman J, Myléus A, Byass P (2014). Undertaking cause–specific mortality measurement in an unregistered population: an example from Tigray Region, Ethiopia.. Glob Health Action..

[25] Byass P (2011). Whither verbal autopsy?. Popul Health Metr.

[26] Roulson J, Benbow EW, Hasleton PS (2005). Discrepancies between clinical and autopsy diagnosis and the value of post mortem histology; a meta–analysis and review.. Histopathology.

[27] Owais AE, Wilson TR, Khan SA, Jaidev J, Renwick I, Mitchell C (2010). Could pre–mortem computerised tomography scans reduce the need for coroners’ post–mortem examinations?. Ann R Coll Surg Engl.

[28] Murray J, Sonnenberg P, Nelson G, Bester A, Shearer S, Glynn JR (2007). Cause of death and presence of respiratory disease at autopsy in an HIV–1 seroconversion cohort of southern African gold miners.. AIDS.

[29] Fligner CL, Murray J, Roberts DJ (2011). Synergism of verbal autopsy and diagnostic pathology autopsy for improved accuracy of mortality data.. Popul Health Metr.

[30] Leitao J, Desai N, Aleksandrowicz L, Byass P, Miasnikof P, Tollman S (2014). Comparison of physician–certified verbal autopsy with computer–coded verbal autopsy for cause of death assignment in hospitalized patients in low–and middle–income countries: systematic review.. BMC Med.

[31] Byass P, Calvert C, Miiro–Nakiyingi J, Lutalo T, Michael D, Crampin A (2013). InterVA–4 as a public health tool for measuring HIV/AIDS mortality: a validation study from five African countries.. Glob Health Action..

[32] Ndila C, Bauni E, Nyirongo V, Mochamah G, Makazi A, Kosgei P (2014). Verbal autopsy as a tool for identifying children dying of sickle cell disease: a validation study conducted in Kilifi district, Kenya.. BMC Med.

[33] Murray CJL, Lozano R, Flaxman AD, Serina P, Phillips D, Stewart A (2014). Using verbal autopsy to measure causes of death: the comparative performance of existing methods.. BMC Med.

[34] Byass P (2014). Usefulness of the Population Health Metrics Research Consortium gold standard verbal autopsy data for general verbal autopsy methods.. BMC Med.

[35] Desai N, Aleksandrowicz L, Miasnikof P, Lu Y, Leitao J, Byass P (2014). Performance of four computer–coded verbal autopsy methods for cause of death assignment compared with physician coding on 24,000 deaths in low–and middle–income countries.. BMC Med.

[36] Murray CJ, Lozano R, Flaxman AD, Vahdatpour A, Lopez AD (2011). Robust metrics for assessing the performance of different verbal autopsy cause assignment methods in validation studies.. Popul Health Metr.

