# Supplementary material for: Comparing verbal autopsy cause of death findings as determined by physician coding and probabilistic modelling: a public health analysis of 54 000 deaths in Africa and Asia
Source: J Glob Health. 2015 Feb 10;5(1):010402. doi: 10.7189/jogh.05.010402 (PMC4337147; doi:10.7189/jogh.05.010402)
Supplement: Online Supplementary Document [file jogh-05-010402-s001.pdf]

Online Supplementary Document

Byass et al. Comparing verbal autopsy cause of death findings as determined by physician coding and probabilistic modelling: a public health analysis of 54 000 deaths in Africa and Asia.

J Glob Health 2015;5:010402

Table s1. Cause-specific mortality fraction (CSMF) ratios InterVA-4:PCVA by WHO 2012 VA cause category and data source, with 99% confidence intervals (shown as significantly less than 1 (< 1); not significantly different (≈) and significantly greater than 1 (> 1))

cause of death	data source											
	Afghanistan		Bangladesh		Ghana		Kenya		South Africa A		South Africa B	
	CSMF ratio	99% CI	CSMF ratio	99% CI	CSMF ratio	99% CI	CSMF ratio	99% CI	CSMF ratio	99% CI	CSMF ratio	99% CI
01.01 Sepsis (non-obstetric)	2.66	≈			1.54	≈	1.22	≈	7.74	> 1	8.10	≈
01.02 Acute resp infect, incl pneumonia	1.21	> 1	12.87	> 1	0.34	< 1	2.20	> 1	3.00	> 1	1.11	≈
01.03 HIV/AIDS related death	60.42	> 1	3.30	≈	1.00	≈	0.64	< 1	1.00	≈	0.43	< 1
01.04 Diarrhoeal diseases	0.86	≈	0.33	< 1	0.24	< 1	0.58	< 1	0.52	< 1	0.26	< 1
01.05 Malaria	0.34	< 1	1.45	≈	0.37	< 1	0.85	< 1	0.36	< 1	1.88	> 1
01.06 Measles	48.89	> 1					2.15	> 1	16.20	≈	15.80	≈
01.07 Meningitis and encephalitis	1.71	> 1	2.66	≈	0.00	< 1	0.28	< 1	0.27	< 1	0.42	< 1
01.08, 10.05 Tetanus							0.29	≈			2.54	≈
01.09 Pulmonary tuberculosis	3.01	> 1	1.79	> 1	1.97	> 1	1.24	> 1	1.69	> 1	4.81	> 1
01.10 Pertussis	9.56	≈					12.50	≈	53.47	> 1	8.20	≈
01.11 Haemorrhagic fever	5.30	≈					6.36	≈	0.69	≈		
01.99 Other and unspecified infect dis	0.25	< 1	0.11	< 1	0.04	< 1	0.74	< 1	0.18	< 1	0.18	< 1
02.01 Oral neoplasms	4.84	≈	3.31	≈	39.41	≈	60.72	> 1	8.80	> 1	1.92	≈
02.02 Digestive neoplasms	0.70	< 1	1.70	≈	7.80	> 1	1.29	> 1	2.86	> 1	1.79	> 1
02.03 Respiratory neoplasms	17.76	> 1	5.31	> 1	43.77	> 1	15.60	> 1	2.35	> 1	11.78	> 1
02.04 Breast neoplasms	0.79	≈	2.30	≈	1.69	> 1	0.32	< 1	2.20	> 1	1.10	≈
02.05, 02.06 Reproductive neoplasms M,F	1.98	≈	1.58	≈	6.47	> 1	0.35	< 1	0.54	< 1	1.27	≈
02.99 Other and unspecified neoplasms	0.76	≈	0.53	≈	0.08	< 1	1.51	> 1	0.94	≈	0.73	< 1
03.01 Severe anaemia	52.94	> 1	4.20	≈	5.40	≈	0.13	< 1	18.92	≈	70.82	> 1
03.02 Severe malnutrition	1.78	> 1	13.61	≈	4.24	≈	0.18	< 1	0.43	< 1	0.76	≈
03.03 Diabetes mellitus	0.30	< 1	1.57	≈	0.13	< 1	0.51	< 1	1.29	≈	0.71	< 1
04.01 Acute cardiac disease	0.49	< 1	0.71	≈	0.73	≈	8.39	> 1	1.37	≈	0.37	< 1
04.03 Sickle cell with crisis					15.98	≈	0.71	≈				
04.02 Stroke	0.88	≈	1.17	≈	0.30	< 1	0.91	≈	0.48	< 1	0.61	< 1
04.99 Other and unspecified cardiac dis	0.35	< 1	2.43	> 1	0.74	< 1	5.95	> 1	0.50	< 1	0.77	< 1
05.01 Chronic obstructive pulmonary dis	1.17	≈	0.97	≈	21.22	≈	0.15	< 1	19.32	> 1	3.49	> 1

05.02 Asthma	1.53	≈	0.57	≈	6.68	> 1	0.76	≈	2.10	> 1	1.34	≈
06.01 Acute abdomen	8.03	> 1	9.84	> 1	8.87	> 1	10.12	> 1	7.17	> 1	99.32	> 1
06.02 Liver cirrhosis	1.31	≈	0.97	≈	0.36	< 1	1.11	≈	0.36	< 1	0.23	< 1
07.01 Renal failure	0.53	≈	1.65	≈	1.30	≈	0.48	< 1	0.36	< 1	0.79	≈
08.01 Epilepsy	0.47	≈	1.12	≈	0.03	< 1	0.26	< 1	0.54	< 1	0.89	≈
98 Other and unspecified NCD	0.29	< 1	0.89	≈	0.05	< 1	38.75	> 1	0.27	< 1	0.04	< 1
10.06 Congenital malformation	0.32	< 1	0.33	≈			0.53	≈	0.14	< 1	0.57	≈
10.01 Prematurity	1.15	≈					0.18	< 1	1.10	≈	0.26	< 1
10.02 Birth asphyxia	10.15	> 1					2.38	> 1	2.23	> 1	0.83	≈
10.03 Neonatal pneumonia	2.63	> 1					24.05	> 1	2.33	> 1	1.88	> 1
10.04 Neonatal sepsis	0.37	< 1					0.17	< 1	2.89	≈	2.43	≈
10.99 Other and unspecified neonatal CoD	0.22	< 1					0.93	≈	0.18	< 1	0.19	< 1
12.01 Road traffic accident	0.90	≈	1.23	≈	1.13	≈	0.83	≈	0.90	≈	1.12	≈
12.02 Other transport accident	0.20	≈			0.33	≈			0.33	≈	0.00	< 1
12.03 Accid fall	0.68	≈	0.33	≈	0.77	≈	2.83	> 1	0.05	≈	0.68	≈
12.04 Accid drowning and submersion	0.77	≈	0.23	≈	0.90	≈	1.81	> 1	0.48	≈	0.75	≈
12.05 Accid expos to smoke, fire & flame	0.46	≈	0.50	≈	0.57	≈	0.82	≈	0.97	≈	1.65	≈
12.06 Contact with venomous plant/animal	0.68	≈	1.00	≈	0.78	≈	0.91	≈	0.05	≈	2.09	≈
12.10 Exposure to force of nature	0.20	≈	0.14	≈	0.09	≈	3.80	≈	0.03	≈	0.11	≈
12.07 Accid poisoning and noxious subs	0.44	≈	1.50	≈	0.15	≈	0.21	< 1	0.86	≈	0.35	≈
12.08 Intentional self-harm	1.43	≈	0.61	< 1	3.86	≈	1.34	≈	0.56	< 1	1.21	≈
12.09 Assault	1.68	> 1	0.54	≈	1.43	≈	1.17	≈	1.06	≈	1.01	≈
12.99 Other and unspecified external CoD	0.09	< 1	0.03	≈	0.04	≈	0.08	< 1	0.48	< 1	0.10	< 1
09.01 Ectopic pregnancy			0.99	≈	1.46	≈	4.90	≈	0.58	≈	3.16	≈
09.02 Abortion-related death	1.67	≈	0.52	≈	0.59	< 1	0.48	≈	0.13	≈	0.52	≈
09.03 Pregnancy-induced hypertension	1.28	≈	1.11	≈	0.17	< 1	1.25	≈	0.60	≈	1.00	≈
09.04 Obstetric haemorrhage	0.87	≈	0.64	≈	1.65	> 1	1.05	≈	3.49	> 1	0.84	≈
09.05 Obstructed labour	0.45	≈	0.14	≈	0.61	≈	2.60	≈				
09.06 Pregnancy-related sepsis	3.64	≈	1.40	≈	0.83	≈	0.81	≈	0.30	≈	0.82	≈
09.07 Anaemia of pregnancy	0.71	≈	0.45	≈	0.12	< 1	17.82	≈	0.98	≈	9.38	≈
09.08 Ruptured uterus			3.85	≈	0.54	≈	0.20	≈			0.33	≈
09.99 Other and unspecified maternal CoD	0.06	≈	0.14	< 1	0.12	< 1	0.57	≈	0.05	< 1	0.12	< 1
99 Indeterminate	2.24	> 1	0.70	< 1	9.68	> 1	3.89	> 1	0.78	< 1	80.21	> 1

Table s2. Cause-specific mortality fractions by WHO 2012 VA cause category and age-sex group

cause		males							females						
		<28d	1-11m	1-4y	5-14y	15-49y	50-64y	65+y	<28d	1-11m	1-4y	5-14y	15-49y	50-64y	65+y
01.01 Sepsis (non-obstetric)	InterVA		0.89	0.25	0.36	0.03	0.11	0.14		0.73	0.33	0.15	0.01	0.06	0.10
	PCVA		0.08	0.04	0.00	0.08	0.06	0.24		0.08	0.00	0.11	0.02	0.00	0.30
01.02 Acute resp infect, incl pneumonia	InterVA		45.65	17.16	12.30	4.07	6.29	8.39		47.27	18.06	12.90	2.84	6.20	8.69
	PCVA	1.38	21.69	9.69	5.41	2.72	3.62	4.95	1.50	23.61	10.08	4.88	2.14	2.22	4.19
01.03 HIV/AIDS related death	InterVA		13.36	27.57	16.17	18.60	14.50	3.81		12.85	28.61	15.01	29.29	20.03	4.84
	PCVA	0.63	20.12	20.94	17.19	45.80	30.68	7.60	2.18	18.38	20.39	17.29	43.74	30.28	5.16
01.04 Diarrhoeal diseases	InterVA	0.31	9.00	6.64	1.98	0.34	0.34	0.51	0.19	8.55	6.34	1.55	0.63	0.48	0.82
	PCVA	0.63	14.51	9.53	3.13	0.82	1.39	2.39	1.50	14.40	8.84	4.21	2.20	1.58	2.99
01.05 Malaria	InterVA		15.25	26.17	13.31	1.60	1.91	1.60		15.65	25.77	12.41	2.15	1.80	2.47
	PCVA	0.85	19.77	23.58	15.26	2.84	3.10	3.48	1.09	20.61	24.29	15.52	3.43	2.80	3.94
01.06 Measles	InterVA		1.97	1.57	0.41					1.82	1.76	0.12			
	PCVA		0.88	0.50	0.12				0.14	0.45	0.72	0.11			
01.07 Meningitis and encephalitis	InterVA	4.06	1.14	0.74	1.72	0.98	0.70	0.24	2.57	1.10	0.31	2.53	0.88	0.48	0.18
	PCVA	0.53	3.49	3.87	5.77	2.22	1.88	0.61	0.96	3.85	4.54	7.21	2.94	1.18	0.76
01.08, 10.05 Tetanus	InterVA		0.03		0.09					0.02					
	PCVA		0.00							0.08	0.08			0.04	
01.09 Pulmonary tuberculosis	InterVA		0.30	2.00	10.42	36.72	28.29	20.33		0.36	1.18	10.55	23.84	21.34	10.55
	PCVA		2.65	4.67	5.05	12.88	12.43	11.23		3.00	4.42	5.43	8.38	11.37	8.89
01.10 Pertussis	InterVA		0.36	0.22						0.71	0.30				
	PCVA														
01.11 Haemorrhagic fever	InterVA			0.01	0.33	0.02						0.07			
	PCVA														0.02
01.99 Other and unspecified infect dis	InterVA		0.30	0.29	2.05	0.84	1.02	1.00		0.34	0.40	2.41	0.29	0.82	0.85
	PCVA	4.13	2.65	1.19	3.13	1.73	1.59	2.22	3.41	2.39	1.71	3.55	2.37	2.05	2.82
02.01 Oral neoplasms	InterVA					0.28	0.21	0.27					0.32	0.06	0.24
	PCVA					0.02	0.06	0.07					0.03	0.07	0.04
02.02 Digestive neoplasms	InterVA					1.86	5.39	4.41					2.16	4.48	3.17
	PCVA				0.12	0.61	2.87	4.17			0.04	0.33	0.65	2.83	3.46

02.03 Respiratory neoplasms	InterVA					1.09	3.21	4.77					1.52	2.09	3.21
	PCVA				0.24	0.10	0.52	0.46			0.04		0.03	0.14	0.32
02.04 Breast neoplasms	InterVA												1.44	0.19	0.54
	PCVA		0.04										0.71	1.22	0.99
02.05, 02.06 Reproductive neoplasms M,F	InterVA					0.15	0.15	0.98					1.98	1.94	2.01
	PCVA						0.52	2.35		0.04			1.09	3.77	3.01
02.99 Other and unspecified neoplasms	InterVA		0.09		0.85	2.10	2.18	4.65		0.06	0.04	0.97	0.51	3.34	4.58
	PCVA		0.15	0.19	2.28	0.57	2.20	5.54		0.12	0.40	2.33	1.78	2.58	4.17
03.01 Severe anaemia	InterVA			0.23	0.15	0.08	0.13	0.58			0.24	0.47	0.17	0.35	0.98
	PCVA			0.04	0.12	0.67	1.04	1.63			0.08		0.64	1.54	2.99
03.02 Severe malnutrition	InterVA		1.46	3.58	2.19	0.23	0.26	0.64		1.01	3.45	1.55	0.10	0.41	0.71
	PCVA		4.53	16.16	3.49	0.04		0.24	0.27	4.99	15.57	2.00	0.03		0.17
03.03 Diabetes mellitus	InterVA		0.02	0.14	0.27	0.58	1.88	3.42		0.00	0.00	0.20	0.30	2.76	3.71
	PCVA		0.08	0.04	0.12	0.56	3.82	5.65		0.04	0.04	0.22	0.76	4.31	4.22
04.01 Acute cardiac disease	InterVA					0.39	0.99	1.24					0.34	0.95	0.87
	PCVA	0.11	0.04			0.32	1.07	1.50					0.53	1.33	1.29
04.03 Sickle cell with crisis	InterVA		0.13	0.54	0.98					0.30	0.74	0.83	0.03		
	PCVA		0.08	0.57	3.13	0.06					0.40	1.88	0.03		
04.02 Stroke	InterVA				0.24	0.82	4.22	4.80				0.35	1.36	4.59	8.26
	PCVA					0.87	5.91	8.56		0.04	0.04	0.11	2.39	6.93	12.77
04.99 Other and unspecified cardiac dis	InterVA				0.24	0.97	4.24	9.78			0.06	1.27	2.84	7.18	13.29
	PCVA		0.12	0.04	1.08	0.91	5.19	9.73		0.08		1.44	2.99	6.93	12.35
05.01 Chronic obstructive pulmonary dis	InterVA				0.08	0.45	1.91	4.39					0.26	1.78	4.83
	PCVA				0.12	0.44	2.32	6.67				0.33	0.20	2.76	7.99
05.02 Asthma	InterVA			0.27	0.73	0.38	1.11	0.99			0.14	2.31	1.88	1.20	1.48
	PCVA		0.12	0.11	0.84	0.32	0.67	0.96		0.04	0.16	0.44	0.57	1.04	0.91
06.01 Acute abdomen	InterVA	0.35	1.29	1.16	3.59	1.90	2.20	3.17	0.39	1.64	1.50	6.52	3.31	2.94	3.28
	PCVA			0.08	0.48	0.26	0.26	0.37			0.08	0.67	0.35	0.25	0.17
06.02 Liver cirrhosis	InterVA		0.14	0.55	1.31	0.40	0.43	1.17			0.32	1.97	0.60	0.53	1.02
	PCVA		0.12	0.04	0.72	0.98	2.32	1.93		0.12	0.12	1.44	1.14	1.51	1.67
07.01 Renal failure	InterVA		0.03	0.05	0.16	0.21	0.78	1.59		0.01		0.56	0.66	0.34	0.84
	PCVA		0.08	0.08	0.84	0.59	1.54	2.15	0.14	0.12	0.16	0.67	0.66	0.61	1.39
08.01 Epilepsy	InterVA		0.14	0.13	2.27	0.46	0.29	0.12		0.11	0.10	1.54	0.20	0.27	0.18

	PCVA	0.32	0.50	0.42	3.37	1.02	0.49	0.33	0.14	0.20	0.40	2.00	0.76	0.43	0.21
98 Other and unspecified NCD	InterVA					0.81	1.30	2.05					0.71	1.03	3.10
	PCVA	0.32	0.84	0.65	1.80	1.29	2.00	3.19	0.41	0.49	0.76	4.99	2.36	1.72	2.61
10.06 Congenital malformation	InterVA	1.41	0.57	0.08					1.30	0.50	0.23		0.01		
	PCVA	2.86	1.15	0.84	0.72	0.04	0.03		2.05	0.97	0.96	1.00	0.03		0.02
10.01 Prematurity	InterVA	11.87							10.44						
	PCVA	16.51	0.65	0.04					16.78	0.57					
10.02 Birth asphyxia	InterVA	24.03							22.70						
	PCVA	8.36	0.19						9.55	0.12	0.04			0.04	
10.03 Neonatal pneumonia	InterVA	30.78							33.47						
	PCVA	8.99							7.37						
10.04 Neonatal sepsis	InterVA	6.74							6.84						
	PCVA	22.75	0.19	0.08					23.87	0.32		0.11			
10.99 Other and unspecified neonatal CoD	InterVA	8.96							7.95				0.01		
	PCVA	23.17	0.08	0.04					21.00				0.01		
12.01 Road traffic accident	InterVA		0.15	0.68	7.75	4.60	1.55	1.07		0.04	0.53	5.60	1.19	0.49	0.26
	PCVA	0.11	0.08	0.73	6.61	4.67	1.51	1.17		0.04	0.56	5.21	1.15	0.65	0.36
12.02 Other transport accident	InterVA														
	PCVA			0.15	0.24	0.62	0.20	0.02		0.04	0.04	0.67	0.13	0.07	
12.03 Accid fall	InterVA		0.11	0.22	0.77	0.09	0.16	0.23			0.12	0.60	0.12	0.16	0.39
	PCVA	0.32	0.15	0.31	0.84	0.08	0.23	0.15		0.04	0.16	0.78	0.16	0.11	0.15
12.04 Accid drowning and submersion	InterVA		0.04	0.88	2.76	0.46	0.06	0.08	0.14	0.08	0.68	1.64	0.13	0.03	
	PCVA		0.15	0.88	3.97	0.28	0.06	0.04	0.14	0.08	0.88	2.77	0.13	0.04	
12.05 Accid expos to smoke, fire & flame	InterVA	0.21	0.42	0.49	0.61	0.14	0.18	0.26		0.28	0.67	0.54	0.16	0.17	0.38
	PCVA	0.11	0.42	0.54	0.84	0.19	0.32	0.15		0.37	0.60	0.89	0.20	0.22	0.29
12.06 Contact with venomous plant/animal	InterVA		0.11	0.38	0.33	0.03	0.09	0.07		0.10	0.24	0.44	0.20	0.07	0.08
	PCVA	0.21	0.12	0.50	0.72	0.07		0.11		0.08	0.20	0.89	0.20	0.14	0.06
12.10 Exposure to force of nature	InterVA			0.11	0.24	0.02		0.02							0.02
	PCVA			0.08	0.60	0.06	0.03	0.04			0.04	0.44	0.07		
12.07 Accid poisoning and noxious subs	InterVA		0.08	0.08	0.30	0.14	0.02	0.06		0.04	0.16	0.22	0.03		0.04
	PCVA		0.35	0.31	0.72	0.35	0.32	0.22		0.12	0.28	0.78	0.11	0.07	0.04
12.08 Intentional self-harm	InterVA				1.26	1.60	0.50	0.28				1.00	1.03	0.34	0.10
	PCVA			0.04	1.32	1.95	0.81	0.52				1.11	0.88	0.32	0.17

12.09 Assault	InterVA		0.08	0.30	2.28	8.90	3.28	0.85		0.04	0.28	0.77	1.07	1.28	0.68
	PCVA		0.04	0.23	1.92	8.32	3.27	0.96		0.04	0.20	0.55	0.89	1.18	0.68
12.99 Other and unspecified external CoD	InterVA		0.08	0.05	1.10	0.21	0.20	0.20		0.15	0.20	0.87	0.00	0.16	0.24
	PCVA	0.95	0.61	0.46	4.09	1.97	0.90	1.56	0.55	0.32	0.80	2.22	0.54	0.65	1.33
09.01 Ectopic pregnancy	InterVA												0.23		
	PCVA												0.15		
09.02 Abortion-related death	InterVA											0.15	0.43		
	PCVA											0.33	0.78		
09.03 Pregnancy-induced hypertension	InterVA												0.72		
	PCVA												0.98		
09.04 Obstetric haemorrhage	InterVA											0.11	2.39		
	PCVA												1.83		
09.05 Obstructed labour	InterVA												0.13		
	PCVA												0.28		
09.06 Pregnancy-related sepsis	InterVA												0.46		
	PCVA												0.53		
09.07 Anaemia of pregnancy	InterVA												0.21		
	PCVA												0.64		
09.08 Ruptured uterus	InterVA												0.09		
	PCVA												0.13		
09.99 Other and unspecified maternal CoD	InterVA												0.26		
	PCVA												1.94	0.04	
99 Indeterminate	InterVA	11.29	6.85	7.45	10.38	7.41	9.94	11.83	14.02	6.22	7.26	11.80	10.50	9.65	13.03
	PCVA	6.77	3.30	2.37	3.61	2.66	4.78	6.86	6.96	3.73	1.91	5.10	5.31	4.99	7.12

Table s3. Cause-specific mortality fraction (CSMF) ratios InterVA-4:PCVA by WHO 2012 VA cause category and age-sex group, with 99% confidence intervals (shown as significantly less than 1 (< 1); not significantly different (≈) and significantly greater than 1 (> 1))

cause	males														females													
	<28d		1-11m		1-4y		5-14y		15-49y		50-64y		65+y		<28d		1-11m		1-4y		5-14y		15-49y		50-64y		65+y	
01.01 Sepsis (non-obstetric)			9.45	>1	4.65	≈	7.05	≈	0.45	≈	1.77	≈	0.59	≈			7.35	>1	17.6 1	≈	1.25	≈	0.43	≈	4.24	≈	0.35	≈
01.02 Acute resp infect, inc pneumonia	0.04	≈	2.10	>1	1.77	>1	2.26	>1	1.50	>1	1.73	>1	1.69	>1	0.04	≈	2.00	>1	1.79	>1	2.63	>1	1.33	>1	2.77	>1	2.07	>1
01.03 HIV/AIDS related death	0.08	≈	0.66	<1	1.32	>1	0.94	≈	0.41	<1	0.47	<1	0.50	<1	0.03	≈	0.70	<1	1.40	>1	0.87	≈	0.67	<1	0.66	<1	0.94	≈
01.04 Diarrhoeal diseases	0.52	≈	0.62	<1	0.70	<1	0.64	≈	0.42	<1	0.25	<1	0.22	<1	0.17	≈	0.59	<1	0.72	<1	0.38	<1	0.29	<1	0.31	<1	0.28	<1
01.05 Malaria	0.06	≈	0.77	<1	1.11	≈	0.87	≈	0.57	<1	0.62	<1	0.46	<1	0.06	≈	0.76	<1	1.06	≈	0.80	≈	0.63	<1	0.64	≈	0.63	<1
01.06 Measles			2.21	>1	3.08	>1	2.59	≈							0.33	≈	3.95	>1	2.42	>1	1.03	≈						
01.07 Meningitis and encephalitis	7.06	>1	0.33	<1	0.19	<1	0.31	<1	0.44	<1	0.37	<1	0.40	<1	2.57	≈	0.29	<1	0.07	<1	0.36	<1	0.30	<1	0.42	<1	0.25	<1
01.08, 10.05 Tetanus			2.34	≈			2.54	≈									0.38	≈	0.20	≈					0.33	≈		
01.09 Pulmonary tuberculosis			0.12	<1	0.43	<1	2.05	>1	2.85	>1	2.27	>1	1.81	>1			0.13	<1	0.27	<1	1.93	>1	2.84	>1	1.88	>1	1.19	>1
01.10 Pertussis			19.6	≈	12.3	≈											36.0	≈	15.9	≈								
01.11 Haemorrhagic fever					1.62	≈	6.53	≈	4.24	≈											2.34	≈					0.33	≈
01.99 Other and unspecified infect dis	0.01	<1	0.12	<1	0.26	<1	0.66	≈	0.49	<1	0.64	≈	0.46	<1	0.02	<1	0.15	<1	0.24	<1	0.69	≈	0.13	<1	0.41	<1	0.31	<1
02.01 Oral neoplasms									11.1	>1	3.14	≈	3.72	≈									10.8	>1	0.90	≈	5.15	≈
02.02 Digestive neoplasms							0.33	≈	3.03	>1	1.87	>1	1.06	≈					0.33	≈	0.14	≈	3.29	>1	1.58	>1	0.92	≈
02.03 Respiratory neoplasms							0.20	≈	10.1	>1	6.01	>1	10.3	>1					0.33	≈			41.1	>1	13.0	>1	9.68	>1
02.04 Breast neoplasms			0.33	≈																			2.03	>1	0.17	<1	0.55	≈
02.05, 02.06 Reproductive neoplasms M,F									29.4	≈	0.31	≈	0.42	<1			0.33	≈					1.82	>1	0.52	<1	0.67	<1
02.99 Other and unspecified neoplasms			0.61	≈	0.09	≈	0.39	≈	3.66	>1	0.99	≈	0.84	≈			0.56	≈	0.14	≈	0.43	≈	0.29	<1	1.29	≈	1.10	≈
03.01 Severe anaemia					4.31	≈	1.18	≈	0.12	<1	0.14	<1	0.36	<1					2.59	≈	9.55	≈	0.26	<1	0.24	<1	0.33	<1
03.02 Severe malnutrition			0.33	<1	0.22	<1	0.63	≈	5.11	>1	19.0	≈	2.59	>1	0.20	≈	0.21	<1	0.22	<1	0.78	≈	3.55	≈	24.0	≈	3.97	>1
03.03 Diabetes mellitus			0.39	≈	2.71	≈	1.82	≈	1.04	≈	0.49	<1	0.61	<1			0.33	≈	0.33	≈	0.94	≈	0.40	<1	0.64	<1	0.88	≈
04.01 Acute cardiac disease	0.33	≈	0.33	≈					1.22	≈	0.93	≈	0.83	≈									0.65	≈	0.72	≈	0.67	≈

04.03 Sickle cell with crisis			1.54	≈	0.95	≈	0.33	<1	0.13	≈							15.7	≈	1.83	≈	0.45	≈	1.16	≈				
04.02 Stroke							5.00	≈	0.95	≈	0.71	<1	0.56	<1			0.33	≈	0.33	≈	2.41	≈	0.57	<1	0.66	<1	0.65	<1
04.99 Other and unspecified cardiac dis			0.14	≈	0.33	≈	0.27	≈	1.07	≈	0.82	≈	1.01	≈			0.20	≈	4.08	≈	0.89	≈	0.95	≈	1.04	≈	1.08	≈
05.01 Chronic obstructive pulmonary dis							0.79	≈	1.03	≈	0.82	≈	0.66	<1							0.14	≈	1.29	≈	0.65	≈	0.60	<1
05.02 Asthma			0.14	≈	2.18	≈	0.88	≈	1.18	≈	1.65	≈	1.04	≈			0.33	≈	0.90	≈	4.74	>1	3.30	>1	1.15	≈	1.62	>1
06.01 Acute abdomen	7.59	≈	68.0	>1	12.4	>1	6.75	>1	7.20	>1	8.05	>1	8.37	>1	6.67	≈	81.6	>1	15.3	>1	9.12	>1	9.35	>1	11.0	>1	18.2	>1
06.02 Liver cirrhosis			1.19	≈	9.83	>1	1.75	≈	0.41	<1	0.19	<1	0.61	<1			0.14	≈	2.42	≈	1.35	≈	0.53	<1	0.36	<1	0.61	<1
07.01 Renal failure			0.48	≈	0.76	≈	0.24	≈	0.37	<1	0.52	<1	0.74	≈	0.33	≈	0.21	≈	0.11	≈	0.85	≈	0.99	≈	0.58	≈	0.61	<1
08.01 Epilepsy	0.14	≈	0.31	≈	0.33	≈	0.68	≈	0.46	<1	0.60	≈	0.38	≈	0.33	≈	0.60	≈	0.29	≈	0.78	≈	0.27	<1	0.64	≈	0.87	≈
98 Other and unspecified NCD	0.14	≈	0.02	<1	0.03	≈	0.03	≈	0.63	<1	0.65	≈	0.64	<1	0.14	≈	0.04	≈	0.03	≈	0.01	<1	0.30	<1	0.60	≈	1.19	≈
10.06 Congenital malformation	0.50	≈	0.50	≈	0.12	<1	0.08	≈	0.11	≈	0.33	≈			0.65	≈	0.52	≈	0.26	<1	0.05	≈	0.33	≈			0.33	≈
10.01 Prematurity	0.72	<1	0.03	≈	0.33	≈									0.62	<1	0.03	≈										
10.02 Birth asphyxia	2.86	>1	0.09	≈											2.37	>1	0.14	≈	0.33	≈					0.33	≈		
10.03 Neonatal pneumonia	3.41	>1													4.51	>1												
10.04 Neonatal sepsis	0.30	<1	0.09	≈	0.20	≈									0.29	<1	0.06	≈			0.33	≈						
10.99 Other and unspecified neonatal CoD	0.39	<1	0.20	≈	0.33	≈									0.39	<1							0.97	≈				
12.01 Road traffic accident	0.33	≈	1.71	≈	0.94	≈	1.17	≈	0.99	≈	1.03	≈	0.91	≈			0.99	≈	0.95	≈	1.07	≈	1.03	≈	0.76	≈	0.73	≈
12.02 Other transport accident					0.11	≈	0.20	≈	0.01	<1	0.07	≈	0.33	≈			0.33	≈	0.33	≈	0.08	≈	0.02	<1	0.20	≈		
12.03 Accid fall	0.14	≈	0.73	≈	0.75	≈	0.92	≈	1.12	≈	0.72	≈	1.51	≈			0.33	≈	0.77	≈	0.79	≈	0.76	≈	1.41	≈	2.47	≈
12.04 Accid drowning and submersion			0.33	≈	1.00	≈	0.70	≈	1.63	≈	0.96	≈	1.76	≈	1.00	≈	0.99	≈	0.78	≈	0.60	≈	1.00	≈	0.92	≈		
12.05 Accid expos to smoke, fire & flame	1.67	≈	0.98	≈	0.92	≈	0.75	≈	0.75	≈	0.57	≈	1.66	≈			0.79	≈	1.12	≈	0.64	≈	0.78	≈	0.81	≈	1.31	≈
12.06 Contact with venomous plant/animal	0.20	≈	0.93	≈	0.76	≈	0.50	≈	0.47	≈	7.22	≈	0.64	≈			1.23	≈	1.17	≈	0.53	≈	0.97	≈	0.56	≈	1.29	≈
12.10 Exposure to force of nature					1.40	≈	0.45	≈	0.38	≈	0.33	≈	0.60	≈					0.33	≈	0.11	≈	0.05	≈			3.00	≈
12.07 Accid poisoning and noxious subs			0.26	≈	0.29	≈	0.46	≈	0.40	<1	0.10	≈	0.31	≈			0.43	≈	0.60	≈	0.33	≈	0.30	≈	0.20	≈	0.96	≈
12.08 Intentional self-harm					0.33	≈	0.96	≈	0.82	≈	0.62	≈	0.56	≈							0.90	≈	1.16	≈	1.06	≈	0.58	≈

12.09 Assault			1.67	≈	1.30	≈	1.18	≈	1.07	≈	1.00	≈	0.89	≈			1.00	≈	1.36	≈	1.36	≈	1.21	≈	1.08	≈	1.00	≈
12.99 Other and unspecified external CoD	0.05	≈	0.16	<1	0.15	≈	0.28	<1	0.11	<1	0.24	<1	0.13	<1	0.11	≈	0.50	≈	0.26	<1	0.41	≈	0.01	<1	0.27	<1	0.19	<1
09.01 Ectopic pregnancy																							1.56	≈				
09.02 Abortion-related death																					0.52	≈	0.55	<1				
09.03 Pregnancy-induced hypertension																							0.74	≈				
09.04 Obstetric haemorrhage																					2.98	≈	1.30	>1				
09.05 Obstructed labour																							0.48	<1				
09.06 Pregnancy-related sepsis																							0.87	≈				
09.07 Anaemia of pregnancy																							0.33	<1				
09.08 Ruptured uterus																							0.70	≈				
09.99 Other/unspecified maternal CoD																							0.14	<1	0.33	≈		
99 Indeterminate	1.66	>1	2.07	>1	3.12	>1	2.85	>1	2.78	>1	2.08	>1	1.72	>1	2.01	>1	1.66	>1	3.77	>1	2.30	>1	1.98	>1	1.93	>1	1.83	>1

Figures A1-A6: CSMFs by InterVA-4 and PCVA, labelled by WHO 2012 VA cause of death category codes (residual categories shown in pink)

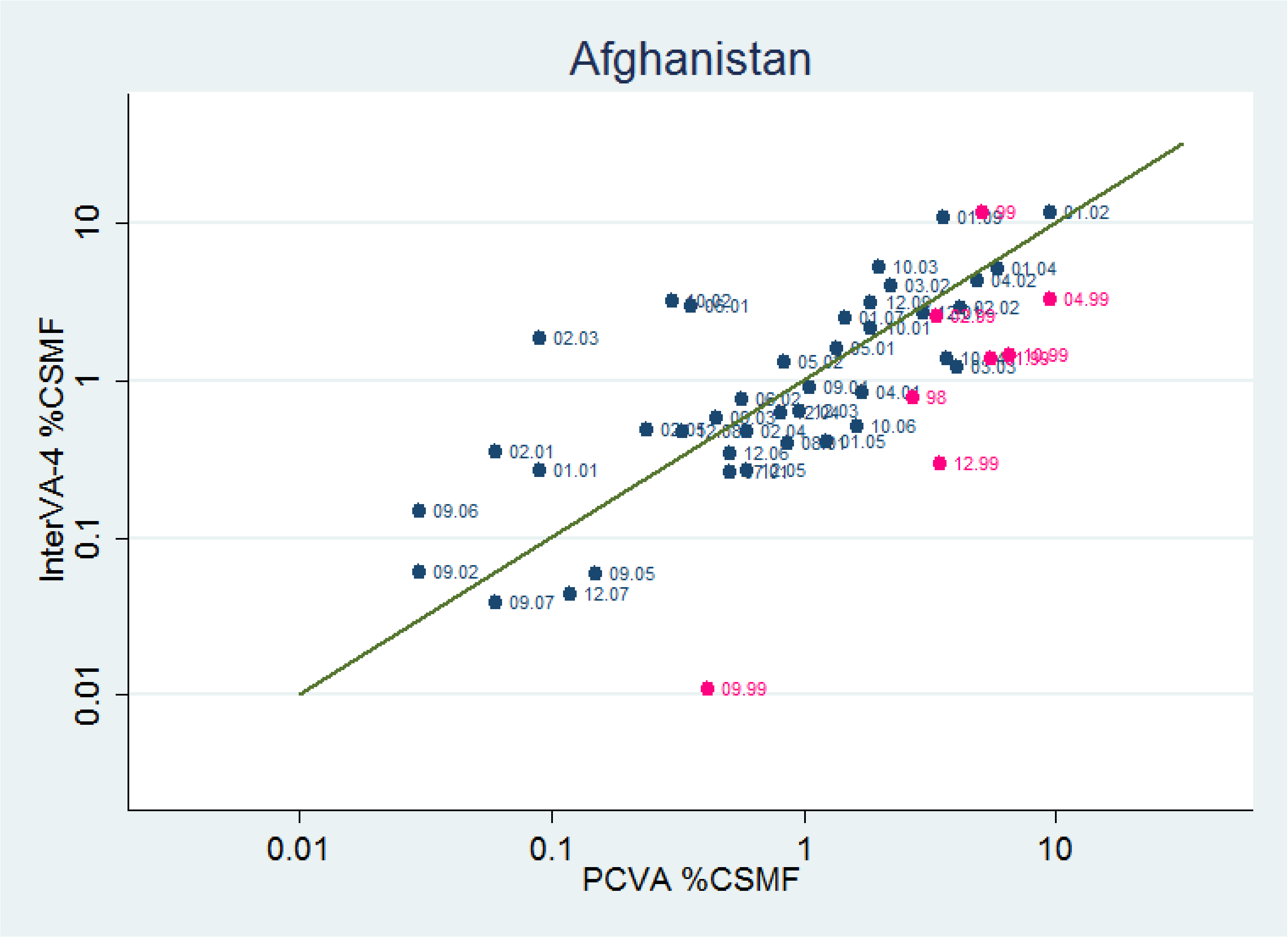

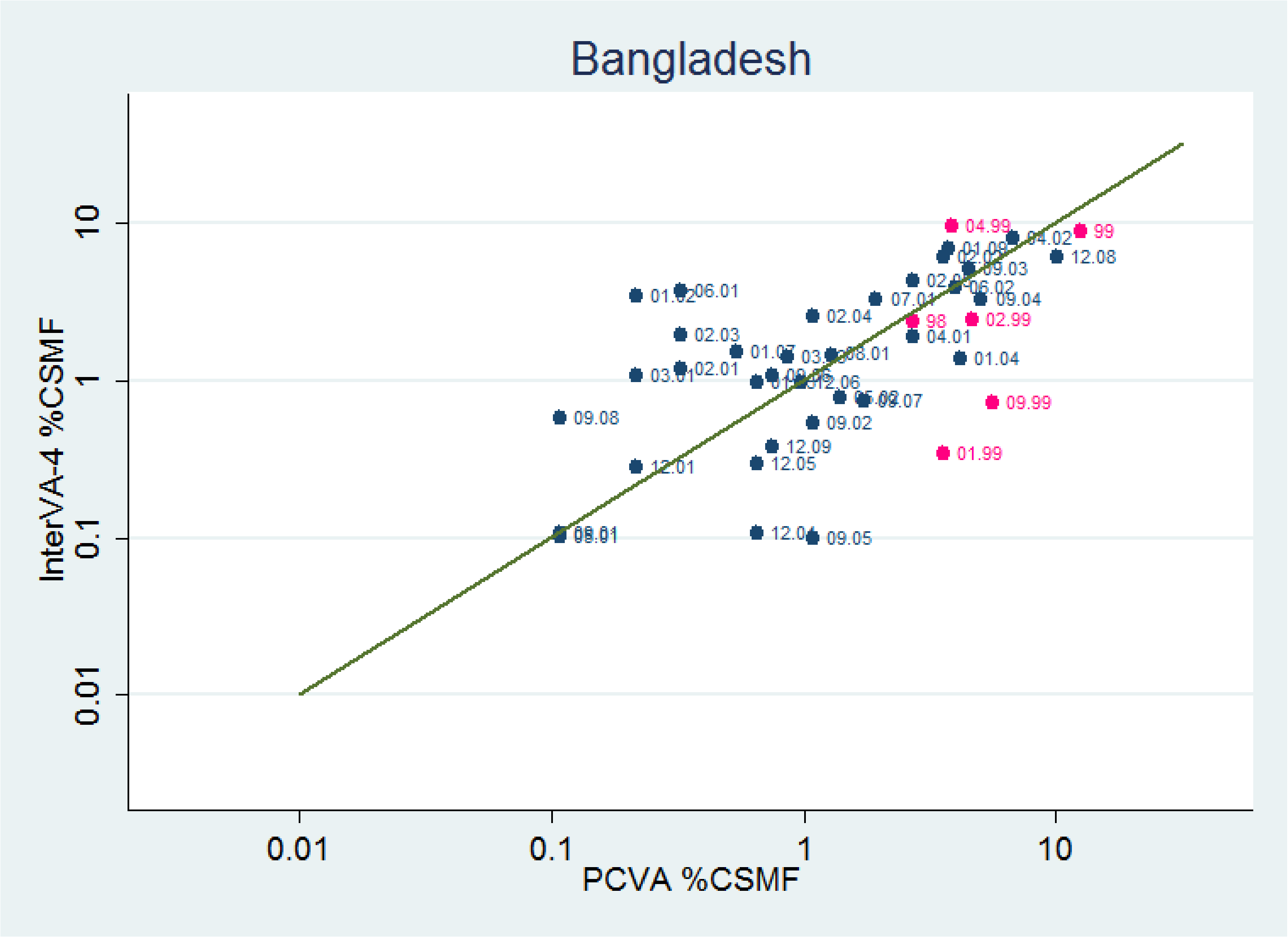

Bangladesh
InterVA-4 %CSMF
PCVA %CSMF
0.01
0.1
1
10
04.99
04.02
99
12.08
09.03
06.01
06.02
09.04
07.01
02.04
98
02.99
02.03
04.01
01.04
08.01
02.01
03.01
12.06
09.07
09.08
09.99
09.02
12.09
12.05
12.01
01.99
09.05

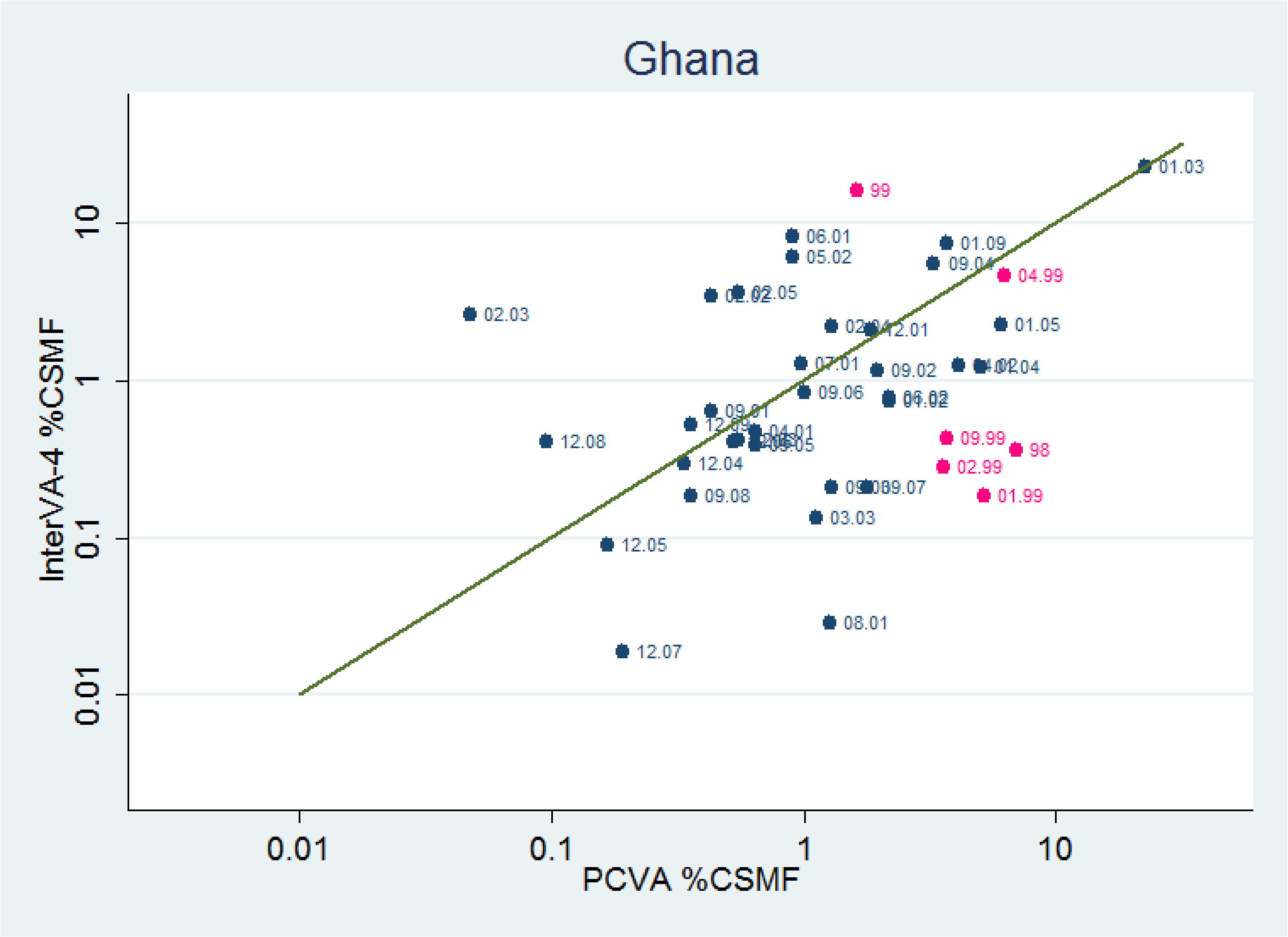

Ghana
InterVA-4 %CSMF
PCVA %CSMF
0.01
0.1
1
10
01.03
99
06.01
05.02
01.09
04.99
02.03
02.05
01.05
12.01
07.01
09.02
09.06
04.01
12.08
12.04
09.99
98
02.99
09.07
09.08
01.99
03.03
12.05
08.01
12.07

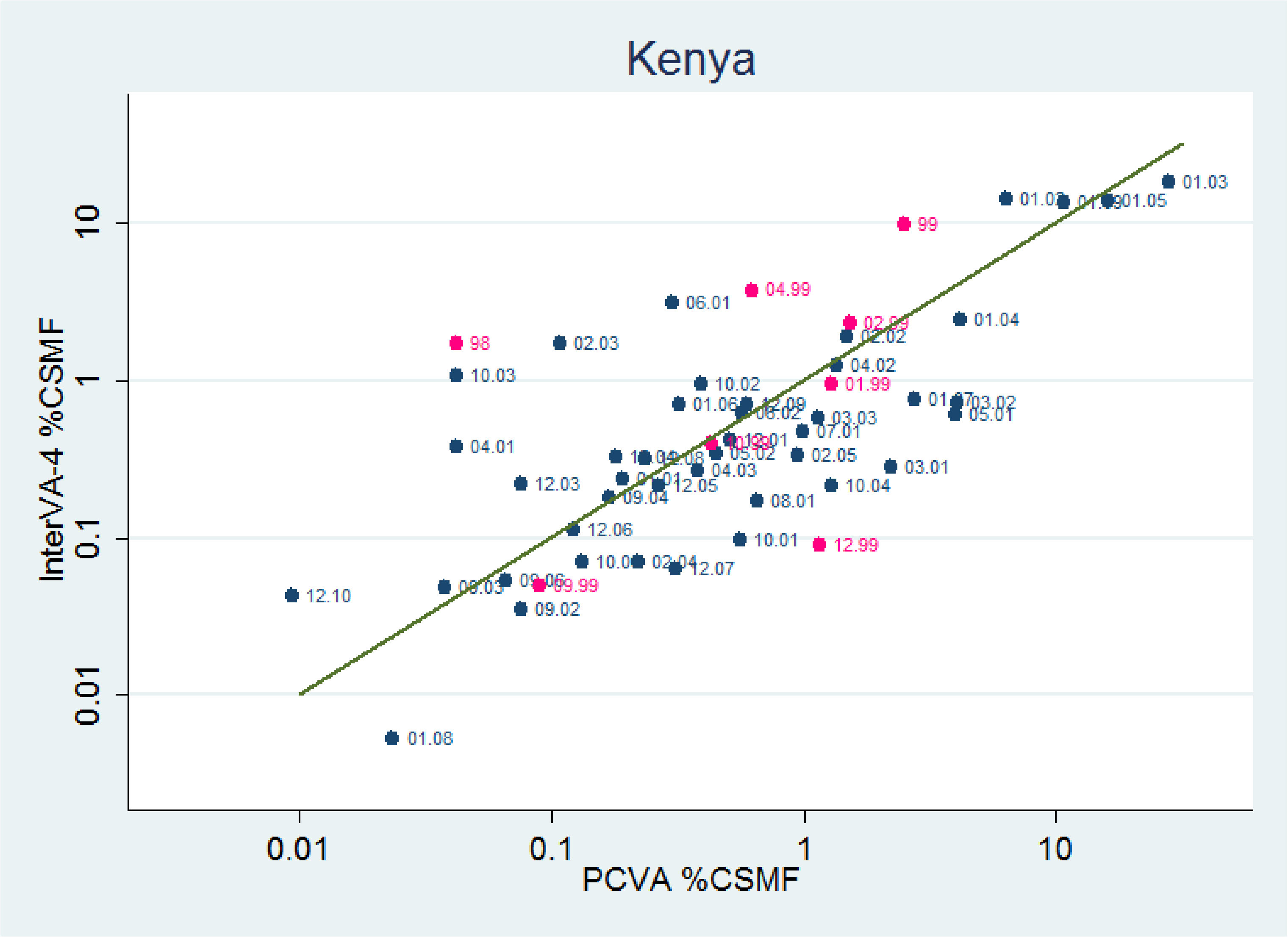
Kenya
InterVA-4 %CSMF
PCVA %CSMF
0.01
0.1
1
10
01.03
01.05
99
04.99
06.01
02.03
98
10.03
01.04
02.02
04.02
10.02
01.99
01.06
03.02
05.01
03.03
07.01
04.01
02.05
03.01
04.03
12.03
12.05
10.04
09.04
08.01
12.06
10.01
12.99
12.07
12.10
09.99
09.02
01.08

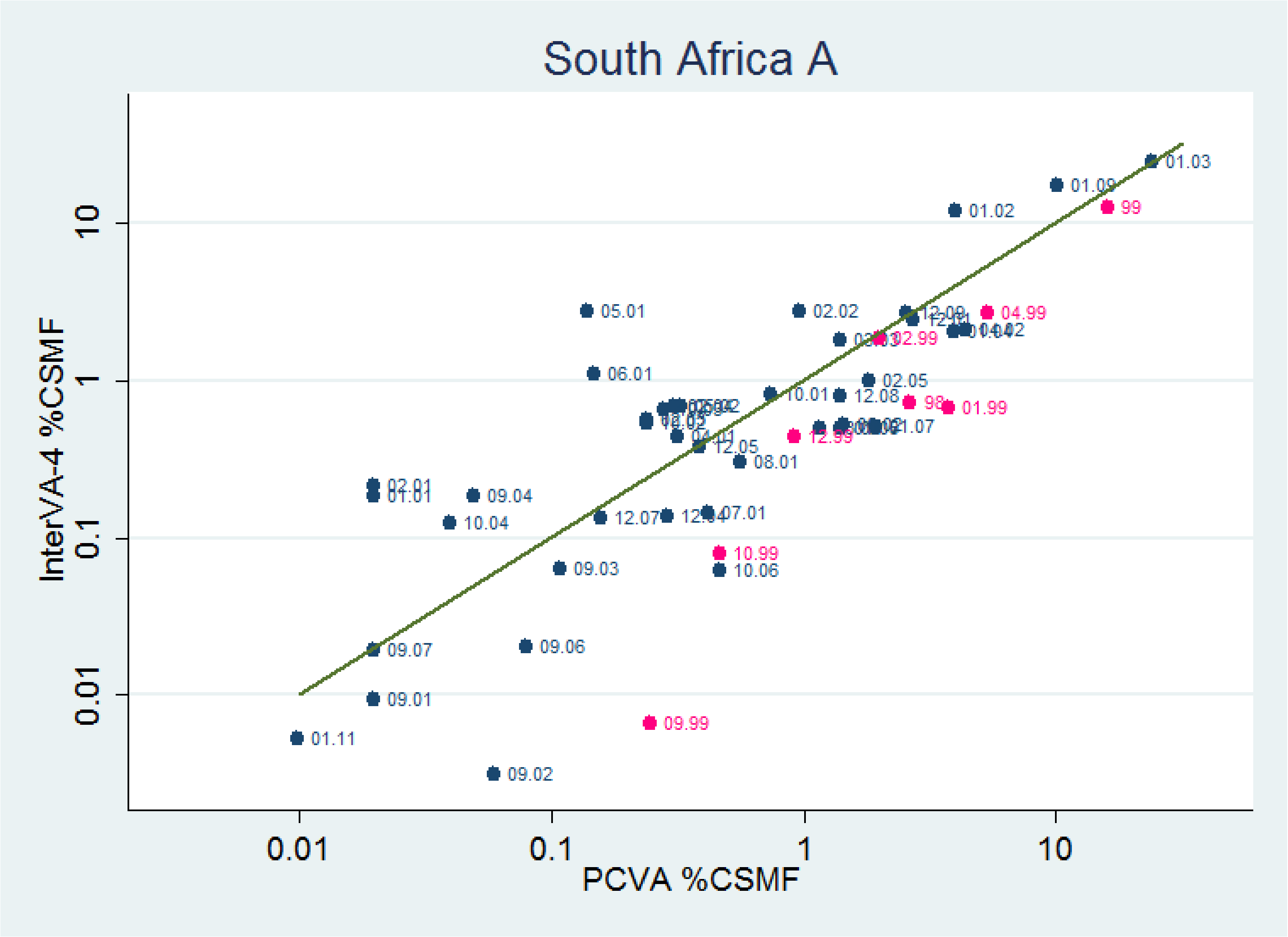
South Africa A
InterVA-4 %CSMF
PCVA %CSMF
0.01
0.1
1
10
01.03
01.09
01.02
99
05.01
02.02
04.99
02.99
06.01
02.05
10.01
12.08
98
01.99
12.99
12.05
08.01
09.04
10.04
12.07
07.01
10.99
09.03
10.06
09.07
09.06
09.01
09.99
01.11
09.02

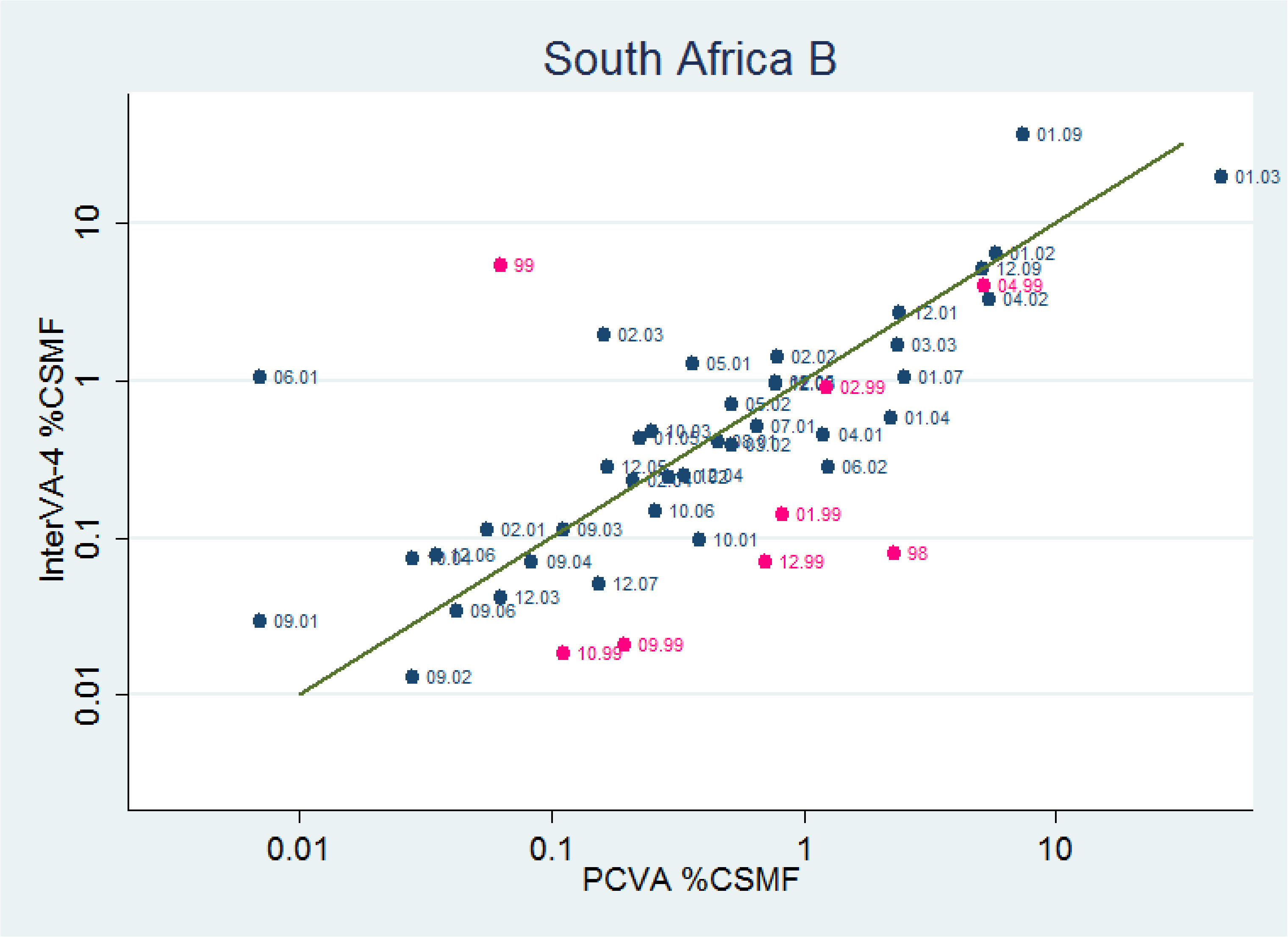

South Africa B
InterVA-4 %CSMF
PCVA %CSMF
0.01
0.1
1
10
01.09
01.03
01.02
12.09
04.99
04.02
99
12.01
03.03
02.03
02.02
05.01
06.01
02.99
01.07
05.02
01.04
07.01
04.01
06.02
10.06
01.99
02.01
09.03
10.01
12.07
09.04
12.03
09.06
12.99
98
09.01
10.99
09.99
09.02
